# Complete transposition of the aorta and pulmonary artery in a Belgian Blue crossbreed calf: A case report

**DOI:** 10.1186/1746-6148-7-22

**Published:** 2011-05-27

**Authors:** Walter Grünberg, Leonie WL van Bruggen, Susanne WF Eisenberg, Erik AWS Weerts, Alan Wolfe

**Affiliations:** 1 Department of Farm Animal Health, Utrecht University, Yalelaan 7, 3584 CL Utrecht, The Netherlands; 2 Department of Clinical Sciences of Companion Animals, Utrecht University, Yalelaan 108, 3584 CM Utrecht, The Netherlands; 3 Veterinary Pathologic Diagnostic Centre, Utrecht University, Yalelaan 1, 3584 CL Utrecht, The Netherlands

## Abstract

**Background:**

Complete transposition of the great arteries is a congenital cardiac malformation occasionally encountered in cattle and other species. The objective of the present report was to provide a detailed clinical, echocardiographic and post mortem description of a calf presenting with this condition.

**Case presentation:**

A 6-week old male Belgian Blue cross-breed calf was examined for respiratory distress and exercise intolerance. The patient was bright, alert and responsive without any neurologic abnormalities but was exercise intolerant, had marked cyanosis, tachycardia, tachypnea, a pansystolic heart murmur as well as a bilaterally palpable thrill over the heart. Arterial blood gas analysis revealed marked hypoxemia (PaO_2 _= 23 mmHg, O_2_sat = 41.1%), mild hypercapnia and compensated respiratory acidosis. Echocardiographic examination revealed a complete transposition of the great arteries in combination with a ventricular septal defect through which blood shunted bidirectionally. Cardiac catheterization confirmed that arterialization of blood of the systemic circulation solely occurred in the right ventricle through blood shunting from the left into the right ventricle. Results of post mortem examination are presented.

**Conclusion:**

Complete transposition of the great arteries is a cyanotic congenital anomaly repeatedly reported in calves that should be considered as differential diagnosis in patients presenting with hypoxemia more severe than commonly encountered with other congenital cyanotic heart conditions. We give a comprehensive summary of the clinical presentation, diagnostic work-up and post mortem examination of a Belgian Blue cross-breed calf with complete transposition of the great arteries

## Background

Congenital heart defects, among which ventricular septal defects (VSD) and atrial septal defects have been reported to be the most common, are occasionally encountered in food animal practice [1,2]. The most frequently encountered congenital heart defects causing cyanosis in cattle are the Tetralogy of Fallot and the Eisenmenger's complex [3]. A congenital malformation of the heart representing the cyanotic cardiac abnormality with the highest incidence in humans (5-7% of all congenital heart anomalies) is the complete transposition of the great arteries (TGA) [4]. This defect is characterized by an aorta arising from the morphologic right ventricle and the pulmonary artery arising from the morphologic left ventricle thus resulting in two separate circulations running in parallel. Complete transposition of the great arteries is listed in several studies investigating the incidence of congenital heart defects in calves [1,2], but although several case reports of patients with TGA in cattle are available these frequently focus on post mortem findings and provide no or very limited detail about clinical presentation of affected animals [5-12]. The objective of the present report was to provide a detailed clinical, echocardiographic and post mortem description of a case of TGA observed in a calf.

## Case presentation

A 6-week old male Belgian Blue cross-breed calf was examined for respiratory distress at the clinic for ruminants of Utrecht University. The calf was reportedly born without complications but showed marked respiratory distress after delivery which improved only mildly in the first hours of life, although it never resolved entirely. Aside from the respiratory distress the calf was otherwise alert. During the first few weeks of life the animal maintained a good appetite and strong suckle reflex, but would tire quickly which meant that it had to interrupt meals repeatedly. Although the growth rate was decreased compared to other calves on the farm the main complaint from the owner was the ongoing labored breathing and rapid exhaustion after excitement.

At the time of presentation to Utrecht University the calf was bright, alert and responsive and in good body condition, although smaller than normal for an animal of its age and breed. The peripheral pulse was strong and equal, with a rate of 140 pulses per minute (reference range 80 - 110). The pulse rate matched the heart rate. Tachypnea (56 breaths per minute, reference range 30-50) and dyspnea were noted. No abnormalities were noted on auscultation of the lungs. Heart auscultation revealed a pansystolic murmur with punctum maximum over the tricuspid valves (V/VI) on the right side of the thorax, and punctum maximum over the pulmonic valves (IV/VI) on the left side of the thorax. A thrill was palpable over the heart that was of equal intensity on the left and right side of the chest. The jugular veins were not distended nor was a jugular pulse present. The mucous membranes were cyanotic. A thorough neurologic exam of the patient using established guidelines [13] did not show any abnormalities other then strong exercise intolerance.

Hematology revealed a PCV of 35% (reference range, 21-37%) and a hemoglobin concentration of 11.9 g/dL (reference range, 8.0 - 15.0 g/dL). Leukogram results were within normal limits. Arterial blood was obtained from an ear artery for blood gas analysis, revealing normocapnia (paCO_2 _= 42 mm Hg, reference range 35 - 44 mm Hg) but marked hypoxemia with an oxygen saturation of 41.1% (reference range > 97%) and a paO_2 _of 23 mm Hg (reference range >79 mm Hg). Similar results were obtained from arterial blood collected from the femoral artery. A blood sample obtained from the jugular vein at the same time showed mild hypercapnia and a compensated respiratory acidosis with a pH of 7.44 (reference range, 7.35 - 7.45) and a pCO_2 _of 53 mm Hg (reference range 35 - 44 mm Hg). The oxygen saturation and pO_2 _in venous blood were 30.6% and 19 mm Hg (reference range 30-40 mm Hg) respectively. Blood lactate concentrations were measured repeatedly at different times in arterial and venous blood and ranged between 0.87 and 1.22 mmol/L (reference range: 0.55-2.22 mmol/L) with venous blood lactate concentrations ranging between 0.1 and 0.3 mmol/L above arterial blood lactate concentrations.

Although the clinical examination and arterial blood gas analysis clearly indicated the presence of a cyanotic heart defect, a Tetralogy of Fallot or an Eisenmenger's complex were considered unlikely differentials as hypoxemia encountered in patients with these conditions are much milder than determined in this animal [14-16].

**Figure 1:**
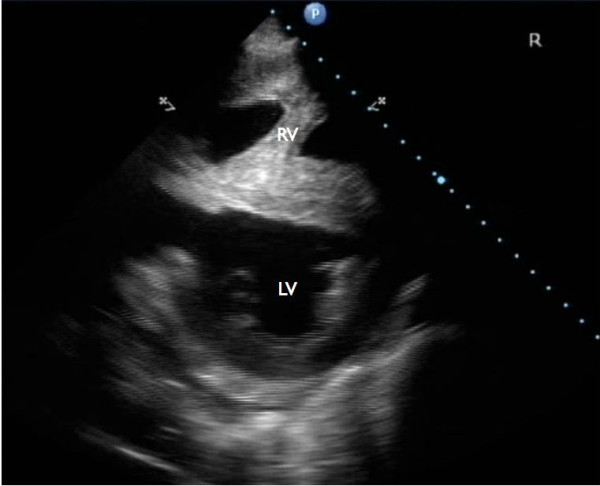
**Short axis, right parasternal view: Enlargement of the right ventricle and flattening of the interventricular septum**. LV: left ventricle, RV: right ventricle.

**Figure 2:**
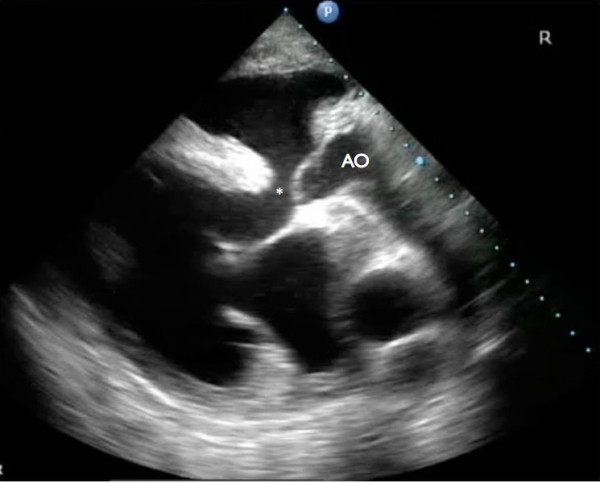
Long axis, right parasternal view: The subvalvular ventricular septal defect (*) as well as the aorta (AO) originating from the right ventricle are visible.

**Figure 3:**
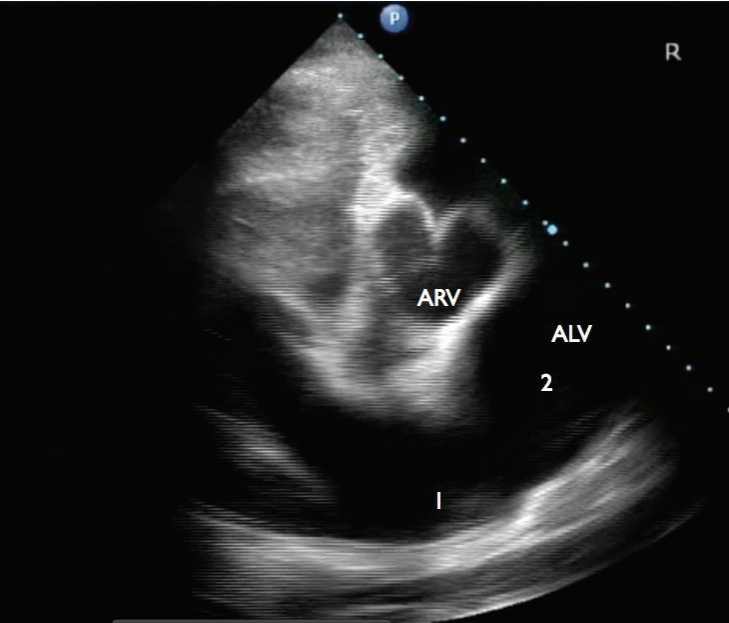
**Short axis, right parasternal view at the level of the heart base: Centrally located is the great artery originating from the right ventricle (ARV)**. The artery originating from the left ventricle (ALV) resembles the normal presentation of a pulmonary artery and its bifurcation. Note the difference in diameter at the level of the bifurcation (1) and the dilatation of this vessel just proximal of this level (2).

**Figure 4:**
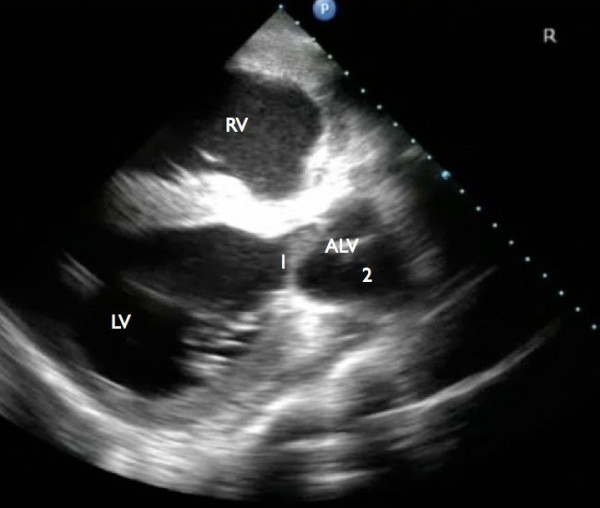
**Further ventral (away from the heart base) and oblique view compared to figure 2a**. Note the artery originating from the left ventricle (ALV) and the difference in diameter of this artery at the level of the valves (1) and distal to these valves (2). RV: right ventricle, LV: left ventricle.

Echocardiographic examination revealed an enlargement of the right ventricle, flattening of the interventricular septum and the presence of a VSD (Figure 1 and 2). The VSD was subvalvular and approximately 1.5-2 cm in diameter. Also, an abnormal position of the left and right ventricular outflow tract was detected, both being slightly off-center. At the level of the heart base, short axis views showed the centrally located artery originating from the right ventricle. At this level the artery originating from the left ventricle could be followed to its bifurcation into two arteries (Figure 3). The diameter of this artery increased a few centimeters after its origin from the left ventricle, resembling a post-stenotic dilatation, and decreased again before its bifurcation (Figure 4). Based on these findings the presence of a functional left outflow tract obstruction was considered.

Color and spectral Doppler revealed bi-directional, turbulent flow through the VSD during systole. Small tricuspid and mitral valvular insufficiencies were also detected. No turbulent flow was detected in the arteries originating from either ventricle. No abnormal flow was detected between the atria. The echocardiograhic examination therefore suggested a complete transposition of the aorta and pulmonary artery. Furthermore a subvalvular VSD with bidirectional blood flow and mild bilateral atrioventricular valve insufficiencies were diagnosed. The presence of a stenosis of the pulmonary artery was considered but could not be confirmed.

Electrocardiographic examination was conducted using a standard bipolar monitor lead (base-apex) revealing a sinus tachycardia as the sole anomaly.

Several weeks after admission, cardiac catheterization using a Swan-Ganz catheter was performed in order to obtain blood from the cranial vena cava, right atrium and right ventricle for blood gas analysis. proper location of the catheter tip was determined based on typical pressure wave tracings for the atrium, ventricle and great artery. Jugular venous blood had a pO_2 _of 15 mm Hg (reference range 35 - 44 mm Hg), an oxygen saturation of 19.8% and a pCO_2 _of 44 mm Hg (reference range 35 - 44 mm Hg). In the right atrium pO_2 _was 16 mm Hg with an oxygen saturation of 21.2%. As the catheter was advanced into the right ventricle a sudden increase of the pO_2 _to 21 mm Hg and of the oxygen saturation to 36.6% was observed.

After completely inserting the catheter (90 cm) into the jugular vein a characteristic arterial pressure curve tracing indicating that the tip of the catheter was located in the great artery exiting the right ventricle was recorded. A wedge pressure tracing identifying the position of the catheter tip in a pulmonary artery branch could not be obtained. Blood sampled from the artery exiting the right ventricle had a pO_2 _of 19 mmHg and an oxygen saturation of 35.2%. Blood collected from the ear artery at the time of cardiac catheterization yielded a pO_2 _of 23 mmHg and an oxygen saturation of 38.9%. These results suggested that arterialization of the blood of the systemic circulation occurred in the right ventricle and that blood from the right ventricle was unlikely to pass through the pulmonary circulation as the oxygen saturation of blood in the right ventricle and arterial blood were similar.

At the time of catheterization the PCV was 48% (reference range 21 - 37%) and hemoglobin concentration was 16.5 g/dL (reference range 8.0 to 15.0 g/dL) revealing a marked increase when compared to values measured at admission.

The calf remained in the clinic until 19 weeks of age when it was humanely euthanized due to a sudden worsening of exercise intolerance and cyanosis over a period of a few days. At the time of euthanasia the calf weighed 94 kg. A normally developed 4-month old Belgian Blue bull calf would be expected to weigh between 170 and 210 kg. A PCV of 65% (reference range 21 - 37%) and a total protein concentration of 6.4 g/dL (reference range 5.9 - 7.5 g/dL) were determined at this time.

**Figure 5:**
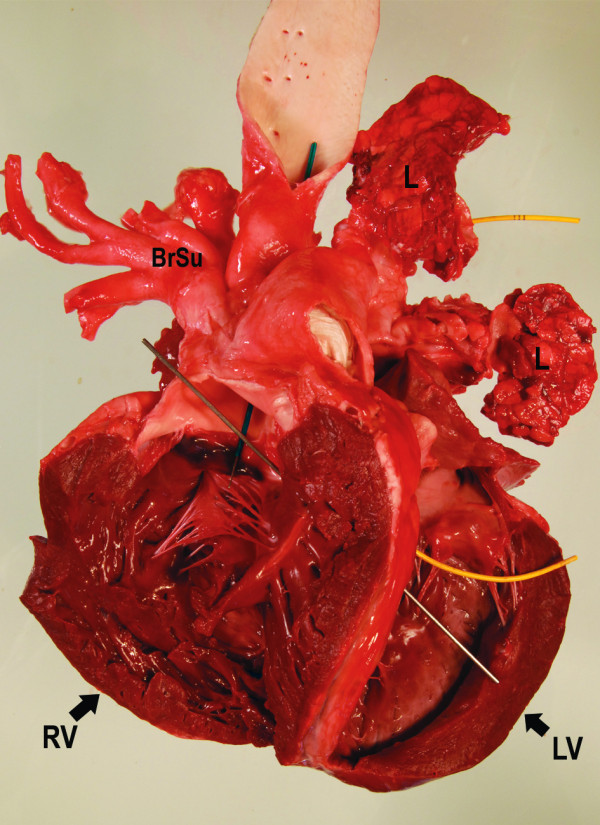
**Heart, view on the opened right ventricle**. The aorta (blue marker) entirely originates from the right ventricle. Note the subvalvular ventricular septal defect (VSD; silver marker) and the marked hypertrophy of the right ventricle. BrSu: Brachyocephalic trunk and left subclavian artery.

**Figure 6:**
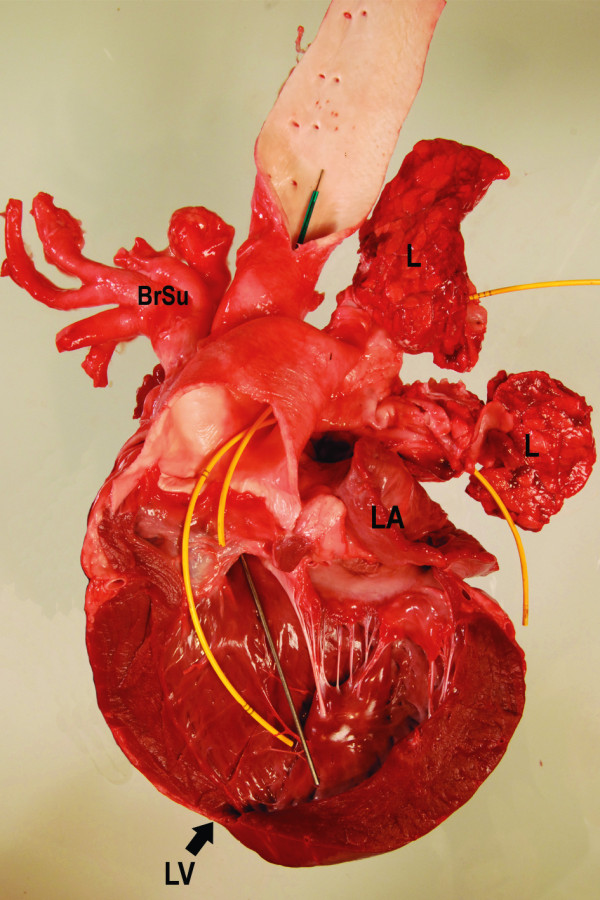
**Heart, view on the opened left ventricle**. The pulmonary artery (yellow markers) originates completely from the left ventricle. Again, note the severe dilatation of the vessel.

**Figure 7:**
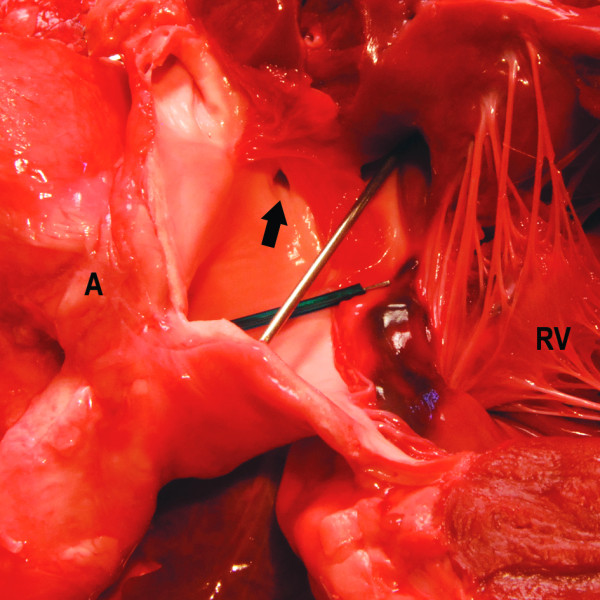
**Heart, detail of the right ventricle with one of the coronary arteries (arrow) originating from the aorta and a closer view on the VSD**.

**Figure 8:**
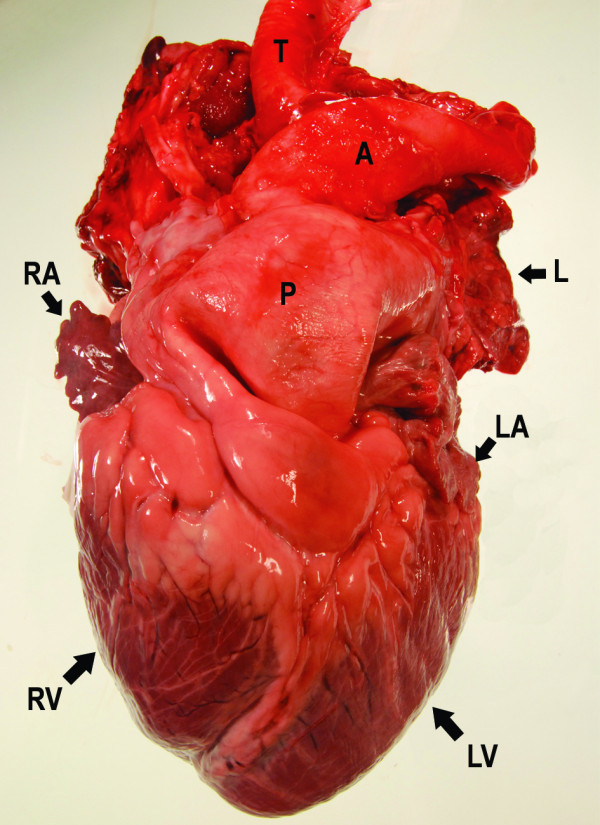
**Heart, intact**. Severe dilatation of the pulmonary artery. A: aorta, P: pulmonary artery, RA: right auricle, LA: left auricle, RV: right ventricle, LV: left ventricle, T: trachea, L: lung tissue.

On post mortem examination the calf was found to be in moderate body condition and showed marked cyanosis of all mucous membranes. Complete transposition of both great vessels was noted, with the aorta exiting from the right ventricle and the pulmonary trunk exiting from the left ventricle (Figures 5 and 6). The width of the aorta at its base was 3.5 cm, and it expanded to 4 cm at its widest point. Coronary arteries were clearly visible arising from around the base of the aorta (Figure 7). The pulmonary artery outflow was 2.5 cm which quickly dilated to 6.5 cm before splitting into the left and right pulmonary branches (Figures 6 and 8). The presence of a morphologic left outflow tract obstruction could not be confirmed on post mortem examination. There was a subvalvular VSD which measured 1.2 × 1.3 cm and the right ventricular wall was markedly hypertrophied with a 1:1 ratio between the left and right free ventricular walls (Figure 5). The ductus arteriosus and foramen ovale were both closed. All other organs were unremarkable. Histology of lung tissue revealed a mild multifocal lymphocytic perivasculitis and moderate multifocal lymphocytic interstitial pneumonia. Lung tissue did not show pulmonary edema, thickening of the wall of pulmonary vessels or any other signs of chronic pulmonary hypertension. Histology of brain tissue did not reveal any abnormalities.

Complete transposition of the great arteries has repeatedly been reported in cattle and is thought to be the result of an abnormal truncus arteriosus development during cardiogenesis with possible genetic or teratogenic etiology [4,17,18]. The incidence is over 95% higher among males than females [19]. Since this malformation causes the systemic and pulmonary circulation to run in parallel it is compatible with life only in combination with at least one more cardiac or extracardial congenital abnormality allowing blood to shunt bidirectionally between the two otherwise completely separated circuits. A left-to-right shunt is required to allow oxygenated blood to enter the systemic blood circuit, whereas a concomitant and volumetrically identical right-to-left shunt is needed to maintain an adequate blood volume in the pulmonary circuit [20].

In the case presented here a VSD was identified as sole connection between pulmonary and systemic circuit. In human patients with TGA a VSD is the second most common concomitant cardiac malformation after a persistent ductus arteriosus with an incidence of approximately 45% [19]. Color and spectral Doppler echocardiography conducted in the patient presented here confirmed the bidirectional turbulent blood flow through the VSD, which obviously allowed sufficient amounts of oxygenated blood to enter the systemic circulation to assure survival, while at the same time maintaining the volume in the pulmonary circulation. A turbulent rather than a unidirectional flow during the systole is suggestive of equal systolic pressures in left and right ventricle. Similar pressure in systemic and pulmonary circulation is commonly observed in human patients with TGA and a large VSD through which pressure at or near the systemic level is transmitted to the pulmonary circuit [20,21].

Tricuspid regurgitation as diagnosed in our patient is also commonly observed in human patients with TGA. Although atrioventricular regurgitation could be caused by a congenital valve abnormality, in most human cases it is considered to be secondary to annular dilatation from right ventricle dysfunction [17]. In the calf presented here all cardiac valves were found to be morphologically normal on post mortem examination, whereas the enlargement of the right ventricle diagnosed echocardiographically would support the hypothesis of ventricular dysfunction and ensuing annular dilatation.

The criterion established in the literature to confirm the diagnosis of TGA is the visualization of the aorta arising from the right ventricle and the pulmonary artery from the left ventricle, either by angiocardiography or echocardiography, as done in the present study [22,23]. Cardiac catheterization in combination with arterial blood gas analysis also provided strong evidence to support the diagnosis of TGA in the case reported here. Whereas the sudden rise of oxygen tension and oxygen saturation in the right ventricle confirmed the presence of a VSD with functional left-to-right shunt, the similar oxygen tension and oxygen saturation in the right ventricle and the ear artery indicated that arterial blood originated from the right ventricle and arterialization solely occurred through pulmonary venous blood shunting from the left to the right ventricle. The inability to obtain a wedge pressure tracing that is normally obtained as the tip of the Swan-Ganz catheter with inflated balloon is advanced into a pulmonary artery branch via the right ventricle further indicated that the catheter was not located in the pulmonary artery.

In the present case extremely low arterial oxygen saturation and paO_2 _values were measured, indicating marked hypoxemia. In humans with acute episodes of hypoxemic hypoxia, as might occur during decompression or at high altitude, an arterial pO_2 _between 40 and 30 mmHg is associated with loss of judgment, euphoria and obtundation whereas values below 25 mmHg, as measured in this patient are associated with coma [24]. However in this calf neurologic deficits such as ataxia, or abnormal reflexes potentially indicating the presence of diffuse cerebral hypoxia were not observed. Histology also showed no abnormalities. Furthermore neither elevated plasma lactate concentrations suggestive of marked hypoxia and ensuing enhanced anaerobic glycolysis were found These findings indicate that neurological tissues can adapt in the presence of congenital chronic severe anoxia.

In an animal with congenital TGA it might be expected that at the time of parturition, and the cessation of perfusion by the umbilical vessels, the animal would suffer from acute hypoxia. However, results from studies looking at oxygen saturation in near-term and neonatal calves suggest differently. For instance, Comline and Sliver [25] reported that, in late-term bovine fetuses, the pO_2 _of the umbilical vein was around 37.6 ± 1.5 mmHg, while Bleul et al. [26] reported that, at the time of birth, the paO_2 _and oxygen saturation values in the arterial blood of calves were 45.3 ± 16.0 mmHg and 64.2% respectively.

Considering that at the time of first examination this patient had a paO_2 _of 23 mmHg and arterial oxygen saturation of 41% it is probable that the change in blood oxygenation at the time of interruption of the blood flow through the umbilical vessels was rather small, explaining the absence of clinical signs commonly observed in patients with acute hypoxemic hypoxia.

The calf in this report appeared to be stable during the first weeks of life, but respiratory distress, exercise intolerance and cyanosis worsened markedly in the last days before euthanasia. However, it did remain bright and alert with a good appetite, without showing any neurologic deficits. The PCV rose from 35% at the time of admission to 48% and later to 65%, while total protein concentration remained constant. This finding is consistent with excessive erythrocytosis observed in patients with chronic hypoxemia as occurs during high altitude disease. In the present case chronic hypoxemia obviously triggered marked erythrocytosis without improving blood oxygenation. The increased amount of unsaturated hemoglobin in blood not only exacerbates cyanosis but also increases blood volume and blood viscosity thereby affecting pulmonary blood flow and thus the pulmonary ventilation-perfusion relationship [27]. This vicious circle is likely to have contributed considerably to the demise of the patient.

In human patients with TGA systemic ventricular dysfunction is a common sequel with advancing age [28] and the most common cause of death in untreated children older than one week [19]. Despite the inherent vulnerability of the morphologic right ventricle to failure, severe hypoxemia as well atrioventricular regurgitation considerably increases the risk of congestive heart failure in these patients.

In cases of TGA combined with a large VSD pulmonary hypertension is another common complication resulting from continuous exposure of the pulmonary circulation to the systemic blood pressure and is likely to contribute to the demise of untreated children having survived the neonatal period. Presence of a pulmonary outflow tract obstruction decreases the risk of pulmonary vascular disease by protecting the pulmonary capillary bed from excessive pressure [29]. Although the presence of a pulmonary outflow tract obstruction could not be ruled out with certitude, lung histology did not show any tissue damage consistent with persistent pulmonary hypertension. It is difficult to assess how much of an affect the interstitial pneumonia seen histologically will have affected this patient clinically.

## Conclusion

Most commonly encountered congenital cardiac anomalies in calves reported in the literature are the Tetralogy of Fallot and the Eisenmenger's complex. Transposition of the great arteries, which is the most common cyanotic cardiac malformation in humans, has received little attention in cattle even though the recent literature suggests a prevalence of this condition only slightly inferior to the prevalence of Tetralogy of Fallot in calves [2]. This case report presents a comprehensive summary of the clinical presentation, diagnostic work-up and post mortem examination of a Belgian Blue cross-breed calf with complete transposition of the great arteries. The present case suggests that TGA should be considered as differential diagnosis in patients presenting with a cyanotic congenital heart defect with more severe hypoxemia than what is commonly encountered with most common congenital heart defects.

## Abbreviations

PCV: packed cell volume; VSD: ventricular septal defect; TGA: transposition of the great arteries

## Competing interests

The author declares that they have no competing interests.

## Authors' contributions

WG contributed to diagnostic work up, coordinated the case and reviewed the literature; LvB conducted echocardiographic examination; SE contributed to the diagnostic work up; AW and EW conducted post mortem examination; all authors contributed to manuscript writing and read and approved the final manuscript.

## References

[B1] Gopal T Leipold HW Dennis SM Congenital cardiac defects in calves Am J Vet Res 1986 47 1120 1121 3717736

[B2] Ohwada K Murakami T Morphologies of 469 cases of congenital heart diseases in cattle J Jap Vet Med Assoc 2000 53 205 209

[B3] Buczinski S Rezakhani A Boerboom D Heart disease in cattle: diagnosis, therapeutic approaches and prognosis Vet J 2010 184 258 263 10.1016/j.tvjl.2009.05.005 19734077

[B4] Digilio MC Casey B Toscano A Calabro R Pacileo G Marasini M Banaudi E Giannotti A Dallapiccola B Marino B Complete transposition of the great arteries. Patterns of congenital heart disease in familial precurrence Circulation 2001 104 2809 2814 10.1161/hc4701.099786 11733399

[B5] Glazier B McAllister H Twomey T Greene H A congenital heart disorder in a calf: Complete transposition of the great vessels Irish Vet J 1981 35 154 158

[B6] Sandusky GE Smith CW Congenital cardiac anomalies in calves Vet Rec 1981 108 163 165 10.1136/vr.108.8.163 7210447

[B7] Kast A Congenital transposition of the aorta and pulmonary artery in cattle Zentralbl Veterinarmed A 1970 17 780 795 4997501

[B8] Koyama H Sako T Mitani S Uchino T Motoyoshi S Nagashima M Yamaguchi A Watanabe H Kaseki K A case of transposition of the great arteries in a heifer Advances in Animal Electrocardiography 1982 15 53 58

[B9] Maehara S Murakami T Hagio M Hamana K Moritomo Y Anatomical observations on 18 bovine cases of complete transposition of the great arteries J Jap Vet Med Assoc 1995 48 79 83

[B10] Nakade T Sonoda M Takahashi K Kurosawa T Ando Y Matsukawa K Matsumoto A Complete transposition of great arteries in a female Holstein calf Journal of the College of Dairying, Hokkaido, Japan 1983 10 121 128

[B11] Vacirca G Boccadoro B Clinical and radiological study of congenital heart disease in cattle Veterinaria 1965 14 395 408

[B12] Vitums A A complete transposition of the origins of the aorta and the pulmonary artery in a calf Cornell Vet 1956 46 282 288

[B13] Constable PD Clinical examination of the ruminant nervous system Vet Clin Food Anim Pract 2004 20 185 214 10.1016/j.cvfa.2004.02.011 15203222

[B14] Rehage J Veltmann P Stadler P Dieckmann M Poulsen-Nautrup C Scholz H Fallot'sche Tetralogie bei einem Kalb - ein Fallbericht Dtsch tierärtzl Wschr 1990 97 532 534 2088708

[B15] Mohamed T Sato H Kurosawa T Okawa S Nakade T Koiwa M Tetralogy of Fallot in a calf: Clinical, ultrasonographic, laboratory and post mortem findings J Vet Med Sci 2004 66 73 76 10.1292/jvms.66.73 14960816

[B16] Vongpatanasin W Brickner E Hillis LD Lange RA The Eisenmenger Syndrome in adults Ann Intern Med 1998 128 745 755 9556469 10.7326/0003-4819-128-9-199805010-00008

[B17] Warnes CA Transposition of the great arteries Circulation 2006 114 2699 2709 10.1161/CIRCULATIONAHA.105.592352 17159076

[B18] Khera KS Fetal cardiovascular and other defects induced by thalidomide in cats Anat Rec 1964 149 299 10.1002/ar.1091490211 124472

[B19] Liebman J Cullum L Belloc NB Natural history of transposition of the great arteries. Anatomy and birth and death characteristics Circulation 1969 40 237 262 4240356 10.1161/01.cir.40.2.237

[B20] Mair DD Ritter DG Ongley PA Helmoholz HF Hemodynamics and evaluation for surgery of patients with complete transposition of the great arteries and ventricular septal defect Am J Cardiol 1971 28 632 640 10.1016/0002-9149(71)90050-6 5120129

[B21] Shaher RM The haemodynamics of complete transposition of the great vessels Brit Heart J 1964 26 343 353 10.1136/hrt.26.3.343 14156085 PMC1018151

[B22] Marino B DeSimone G Pasquini L Giannico S Marceletti C Ammirati A Guccione P Boldrini R Ballerini L Complete transposition of the great arteries: Visualization of left and right outflow tract obstruction by oblique subcostal two-dimensional echocardiography Am J Cardiol 1985 55 140 1145 10.1016/0002-9149(85)90651-4 3984891

[B23] Sleeper MM Palmer JE Echocardiographic diagnosis of transposition of the great arteries in a neonatal foal Vet Radiol Ultrason 2005 46 259 262 10.1111/j.1740-8261.2005.00035.x 16050286

[B24] Butterworth RF Siegel GJ, Agranoff BW, Albers RW, Fisher SK, Uhler MD Metabolic encephalopathies Basic Neurochemistry, molecular, cellular and medical aspects 1999 6 Philadelphia Lippincott-Raven

[B25] Comline RS Silver M A comparative study of blood gas tensions, oxygen affinity and red cell 2,3 DPG concentrations in foetal and maternal blood in the mare, cow and sow J Physiol 1974 242 805 826 4475693 10.1113/jphysiol.1974.sp010735 PMC1330663

[B26] Bleul U Lejeune B Schwantag S Kahn W Blood gas and acid-base analysis of arterial blood in 57 newborn calves Vet Rec 2007 161 688 691 10.1136/vr.161.20.688 18024924

[B27] Leon-Velarde F Villafuerte FC Richalet JP Chronic mountain sickness and the heart Prog Cardiovasc Dis 2010 52 540 549 10.1016/j.pcad.2010.02.012 20417348

[B28] Graham TP Bernard YD Mellen BG Celemajer D Baumgartner H Cetta F Conolly HM Davidson WR Dellborg M Foster E Gersony WM Gessner IH Hurwitz RA Kaemmerer H Kugler JD Murphy DJ Noonan JA Morris C Perloff JK Sanders SP Sutherland JL Long term outcome in congenitally corrected transposition of the great arteries: a multi-institutional study J Am Coll Cardiol 2000 36 255 261 10.1016/S0735-1097(00)00682-3 10898443

[B29] Viles PH Ongley PA Titus JL The spectrum of pulmonary vascular disease in transposition of the great arteries Circulation 1969 40 31 41 5802510 10.1161/01.cir.40.1.31

